# *Drosophila melanogaster* as a Tool for Amyotrophic Lateral Sclerosis Research

**DOI:** 10.3390/jdb10030036

**Published:** 2022-08-30

**Authors:** Krupa N. Hegde, Ajay Srivastava

**Affiliations:** 1 Gatton Academy of Mathematics and Sciences at Western Kentucky University, Bowling Green, KY 42101, USA; 2 Department of Cell Biology, Yale College, New Haven, CT 06520, USA; 3 Department of Biology, Western Kentucky University, Bowling Green, KY 42101, USA

**Keywords:** ALS, *Drosophila*, genetics

## Abstract

Reliable animal model systems are an integral part of biological research. Ever since Thomas Hunt Morgan won a Nobel Prize for genetic work done using the fruit fly (*Drosophila melanogaster*) as a model organism, it has played a larger and more important role in genetic research. *Drosophila* models have long been used to study neurodegenerative diseases and have aided in identifying key disease progression biological pathways. Due to the availability of a vast array of genetic manipulation tools, its relatively short lifespan, and its ability to produce many progenies, *D. melanogaster* has provided the ability to conduct large-scale genetic screens to elucidate possible genetic and molecular interactions in neurodegenerative diseases such as Alzheimer’s disease, Parkinson’s disease, Huntington’s Disease, and Amyotrophic Lateral Sclerosis (ALS). With regards to ALS, many of the gene mutations that have been discovered to be linked to the disease have been modeled in *Drosophila* to provide a look into a detailed model of pathogenesis. The aim of this review is to summarize key and newer developments in ALS research that have utilized *Drosophila* and to provide insight into the profound use of *Drosophila* as a tool for modeling this disease.

## 1. Introduction 

Thomas Morgan’s pioneering of the fruit fly as a model organism helped establish the baseline use of *Drosophila melanogaster* in biological research. Morgan and his team’s use of the fruit fly to define genetic principles catapulted this species to a famed model organism. This was compounded when Hermann Muller formulated the use of balancer chromosomes to maintain stocks with mutations on single chromosomes over many generations. Together, these developments cemented *Drosophila* as an ideal organism for genetic studies. Over time, various other genetic tools have been devised to study the role of genes in *Drosophila* further, including the Q-system [1], UAS-gal4 system [2] MiMIC [3], and CRISPR/Cas9 [4]. The prominence of using *Drosophila* for research was highlighted when Nusslein-Volhard, Wieschaus, and Lewis won the Nobel Prize for using *Drosophila* to study the roles of genes in embryonic development. The usefulness of *Drosophila* was further highlighted when the sequence of the *Drosophila* genome was released in March 2000. All of these together established *Drosophila* as a legitimate model for human health and disease studies, especially since the finding that approximately 77% of known human disease genes have orthologues in the fruit fly genome was unveiled [5]. Prominently, *Drosophila* has been used heavily in studying neurodegenerative diseases, including Huntington’s Disease, Parkinson’s disease, Alzheimer’s (Table 1), and Amyotrophic Lateral Sclerosis (ALS). 

Within all of these diseases, *Drosophila* has proven to be useful in identifying possible genetic causes as well as modeling disease phenotypes. The model organism has also been used to test potential therapeutics [6].

This review focuses primarily on new developments in genetic causes and mechanisms of disease relating to the most prevalent genes found in ALS patients and how *Drosophila* has aided in this research. Through examining some major players in ALS research and the role that *Drosophila* played in elucidating the characteristics of possible genetic factors and molecular mechanisms, we aim to provide a summary of the history and importance of *Drosophila* in ALS research along with highlighting recent developments in the field.

## 2. Amyotrophic Lateral Sclerosis—An Introduction

Amyotrophic lateral sclerosis is a currently incurable disease primarily characterized by the progressive degeneration of motor neurons. Physiological symptoms include localized muscle weakness, speech disturbances, fatigue, and possible fasciculations and cramps. Eventually, the progressive degeneration leads to respiratory failure—the main cause of death in ALS [15]. The prognosis for the disease is quite poor, with the median survival time from onset and detection to death ranging from 20 to 48 months. However, 10%–20% of ALS patients survive longer than 10 years after onset, which highlights the individuality of the disease [16]. ALS is subdivided into two main types: familial ALS (fALS) and sporadic ALS (sALS) (Table 2). Current research in ALS is focused on a number of different areas, including the mechanism of pathogenesis, genetic causes, and finding a treatment regimen (Table 3).

## 3. An Initial Player: The Role of SOD1 in ALS

Superoxide dismutase 1 (SOD1) was the first protein reported to be associated with ALS [31] and current data shows that a variety of different mutations in SOD1 account for approximately 12–15% of fALS cases and 1% of sALS cases [32]. SOD1 normally exists as a homodimer that forms a heterodimer with a copper chaperone for SOD1 (CSS) for copper transfer.

By 1995, a fly model was developed utilizing mutants created by mutagenizing genetically marked chromosomes, SOD^F^ or SOD^S^, with ethyl methanesulfonate, γ irradiation, or hybrid dysgenesis. This mutagenesis associated missense mutations located in SOD in *Drosophila* with lesser SOD enzymatic activity and suggested a dimer disequilibrium model in which SOD activity in mutants is lowered through the entrapment of wild-type (WT) subunits into heterodimers [33]. Additionally, researchers showed that the overexpression of SOD1 in the motor neuron extended the *Drosophila* lifespan and rescued the lifespan of a mutant that does not express any of the three *Drosophila* SODs [34].

Somewhat contrastingly, this association of SOD1 to *Drosophila* lifespan was shown to be related to ubiquitous overexpression of SOD1 and not to selective overexpression in the nervous system or muscle cells [35]. Surprisingly, neither pan-neuronal nor pan-glial overexpression of human SOD1 extended the lifespan of *Drosophila*; however, ubiquitous knockdown of SOD1 through RNAi resulted in reduced lifespan in flies. Furthermore, it was determined that the expression of WT or disease-linked mutants of human SOD1 (hSOD1) selectively in motor neurons brought about climbing defects, defective neural circuit electrophysiology, and accumulation of hSOD1 proteins in motor neurons [36]. These studies highlight the versatility of *Drosophila* models.

SOD1’s association with ALS in *Drosophila* was recently explored with a knock-in model, in which four human ALS-causing SOD1 mutations were engineered into the endogenous locus of *Drosophila* SOD1 (dSOD1). Doing this through homologous recombination is a testament to the versatility of *Drosophila,* especially when it comes to investigating the roles that genes play in various disease mechanisms. This knock-in model achieved ALS-like phenotypes without the overexpression of dSOD1. It resulted in flies exhibiting neurodegeneration, locomotor deficits, and the characteristic shortened lifespan similar to that seen in ALS. Furthermore, muscular atrophy and denervation were shown in two of these mutants, consistent with the characteristic symptoms in human ALS patients [37]. Further research using this *Drosophila* knock-in model has surmised the possibility that nonmotor neurons are also implicated in ALS. Both early and late-stage dSOD1 mutant flies were shown to have motor defects that can be mitigated by bone morphogenic protein signaling present within some interneurons [38].

Though *Drosophila* has served as a model organism for multiple studies showing various outcomes of the knockdown or overexpression of SOD1, a specific mechanism of disease has yet to be found. A *Drosophila* transgenic model expressing zinc-deficient hSOD1 was used to examine the cellular toxicity of the SOD1 mutation. The study found that the zinc-deficient mutants experienced a decrease in physical activity and deterioration of mitochondrial structure, which suggests a disease mechanism of zinc-deficient SOD1 mutations causing mitochondrial dysfunction [21]. *Drosophila* models have allowed for invaluable research into the SOD1 gene and its toxicity in relation to ALS.

## 4. Alsin’s Association with ALS

A second protein, Alsin, was found to be associated with ALS in 2001 [39]. It is encoded by the *ALS2* gene. The protein is a guanine nucleotide exchange factor for the GTPase Rab5 [40]. A *Drosophila* model for Alsin-ALS was developed using the *Drosophila* ortholog, dALS2. Consistent with ALS in humans, the *Drosophila* mutants lacked approximately 30% of their coding sequence and showed a significant reduction in locomotion when compared to WT flies [41]. This locomotion reduction was rescued by ubiquitous WT dALS2 overexpression, which suggests a loss-of-function cause, shown through a *Drosophila* model [42]. 

## 5. The Role of VAP-B in ALS

A novel mutation associated with ALS was found in 2004 in a Brazilian family [43]. in protein VAPB (VAMP (Vesicle Associated Membrane Protein) Associated Protein B). VAPB, a member of the VAP (vesicle associated membrane protein-associated protein) family, plays a role in the unfolded protein response and is activated when misfolded proteins accumulate in the ER. An elevation of ER stress has been recorded in the motor neurons of ALS patients, indicating its possible role in the pathogenesis of ALS [44].

In *Drosophila*, the VAPB homolog dVAP-33 controls boutons at the neuromuscular junction (NMJ) and causes postsynaptic glutamate receptor clustering. The UAS/Gal4 system was used in *Drosophila* to alter the expression of dVAP-33A in neurons and showed significant changes in the neuromuscular junction. Both hypomorphic and null mutations in dVAP-33A mutants showed a severe decrease in bouton numbers and an increase in bouton size while overexpression induced an increase in bouton number with a decrease in size. Mutants also displayed changes in glutamate receptors found at the NMJ with regard to subunit abundance and cluster size. This could suggest a mechanism of toxicity dependent on changes at the NMJ. These results found in *Drosophila* could prove to be highly corollary to humans due to the homologous nature between the human and the *Drosophila* VAPB protein, shown by the fact that the loss of dVAP-33A phenotypic changes are rescued by targeting the expression of hVAPB in *Drosophila* neurons [45].

More recently, a *Drosophila* model was established to investigate VAMP’s role in the ER and its association with ALS. dVAP-33A null mutant larvae were observed to have a substantial decrease in crawling speed when compared to genomic rescue controls, and it was found that a loss of dVAP-33A led to an accumulation of Atg8a-II in the brain, fat body, and salivary glands. Atg8a is associated with autophagy in *Drosophila*, and a transmission electron microscopy analysis of dVAP-33A mutant flies showed a dramatic accumulation of autophagosomes, lysosomes, and autolysosomes, thus proposing an association between autophagy and the mechanism of disease. More specifically, *Drosophila* data showed that a failure to connect the ER to the Golgi when VAPs are lost leads to an expansion of endosomes causing an accumulation of dysfunctional lysosomes and a failure of autophagic lysosomal degradation that leads to ER stress and possibly to ALS [22].

Another possible mechanism of disease with regards to VAPB was illustrated in *Drosophila* using loss of dVAP-33A mutants. These mutants were shown to have severe mitochondrial defects in adult muscles, suggesting a possible relation to mitochondrial dysfunction [24]. A mechanism of mitochondrial dysfunction would be consistent with previously mentioned studies regarding SOD1 research, illustrating common pathways between the two.

Though possible mechanisms of disease relating to VAMP have been presented, contrasting research has been found in *Drosophila* models concerning a method of toxicity. Potential methods have been thought to primarily include toxic gain of function, haploinsufficiency, and dominant negative effects. Data collected from *Drosophila* shows that VAP mutant proteins form ubiquitinated aggregates that could possibly induce aggregation of WT protein and render it inactive [46], which suggests a dominant negative mechanism, while other data depicts that neuronal expression of pathogenic VAP induces aggregate formation and depletes the WT protein from normal localization, but those effects were not observed when the WT protein was overexpressed, indicating a toxic gain of function mechanism [45]. Despite this conflicting data regarding the specificities of the mechanism of toxicity, *Drosophila* models of VAMP have allowed for the characterization of this mutation in regard to ALS.

## 6. *Drosophila’s* Role in Developing an Understanding of TDP-43 and ALS

TAR DNA binding protein 43 (TDP-43) is an RNA/DNA binding protein that has been linked to RNA-related metabolism and processing. In 2006, TDP-43 was found to be a component of the insoluble inclusions in the brains of patients with ALS, and new research has found that a significant portion of ALS cases involve TDP-43 aggregation [47]. Many studies have utilized *Drosophila*, and its ortholog of TDP-43 TBPH, to help characterize this gene and its specific role in the pathogenesis of ALS, including characterizing whether or not the pathogenesis is caused by a gain or loss of function in the protein.

A model created in 2010 used transgenic flies that were developed using the UAS/gal4 system that expressed hTDP-43 in varying neuronal subpopulations. These flies expressed human TDP-43 (hTDP-43) and mutant hTDP-43 lacking its amino-terminal domain [48]. It was found that flies expressing hTDP-43 in the eyes showed loss of ommatidia and showed progressive degeneration with aging. Interestingly, though, mutant hTDP-43 flies did not express this, nor did they express the ommatidia disorganization and large vacuoles present in the eyes that their WT counterparts did. Expressing hTDP-43 in mushroom bodies showed significant axon loss and neuronal death and expressing the gene in motor neurons caused axon swelling and motor neuron loss together with functional deficits, highlighting the possible importance of hTDP-43 in ALS pathogenesis [48].

A potential model of TDP-43 toxicity was proposed using *Drosophila.* It was found that in adult flies, the accumulation of hTDP-43 in the cytoplasm is sufficient to cause degeneration, which is consistent with models of toxicity involving mislocalization seen in other ALS-related genes. It was also observed that the knockdown of TBPH (the drosophila homolog of TDP-43) caused no phenotypic change alone, suggesting a toxic gain of cytoplasmic TDP-43 could be a mechanism of pathogenesis. This is not certain, however, as a loss of nuclear TDP-43 function could also play a role in pathogenesis [49].

Another model of toxicity proposed using *Drosophila* has been one of mitochondrial dysfunction. The eye mitochondria of transgenic flies expressing hTDP-43, induced via a heat shock through Elav-Gal4 pan-neuronal driver, showed a significant decrease in size when compared to controls. Furthermore, 85% of mitochondria in the photoreceptors of hTDP-43 expressing flies exhibited swollen or vesicular cristae. This mitochondrial cristae damage was consistent with those detected in the brain tissues of hTDP-43 proteinopathy patients [25], which shows the usefulness of *Drosophila* as a model to study this gene. The mitochondrion is known to be a source for the production of reactive oxygen species (ROS), with mitochondrion dysfunction leading to the accumulation of ROS, which in turn affects neuronal survival and function. Elevated mitochondrial ROS levels were measured in hTDP-43 expressing motor neurons in *Drosophila* using confocal imaging, which indicates that hTDP-43 expression in motor neurons leads to mitochondrial dysfunction and possible oxidative stress [25]. 

*Drosophila* has also been a model organism to investigate possible modifiers of hTDP-43. It was found that a group of genes involved in mitochondria and oxidative processes, such as uncoupling protein 4b (involved in the uncoupling components in the electron transport chain in reducing oxidative phosphorylation), were altered in flies expressing hTDP-43, an idea in line with the oxidative mechanisms of pathogenesis proposed in various SOD1 and VAMPB models [50]. Another main group of genes were found to be related to the regulation of the cell cycle. Interestingly, a large number of genes affected by TDP-43 expression were targets of the Notch signaling pathway, including the Notch target *Hey*. It was found that mutations that diminished Notch activity extended the lifespan of hTDP-43 expressing flies, which suggests the notion that Notch activation is deleterious in this model [50]. Using *Drosophila* to determine various modifiers of TDP-43 is integral to understanding how the gene functions within ALS pathogenesis.

## 7. The Role of FUS in the Pathogenesis of ALS

In 2009, a missense mutation in a new gene was found to be related to ALS cases by two separate groups independently [51,52]. This gene, FUS/TLS (fused in sarcoma, translated in liposarcoma—often referred to as FUS) is a predominantly nuclear protein that is implicated in DNA repair and transcription regulation, RNA splicing, and export to the cytoplasm [52]. It is an RNA-binding protein that has also been implicated in various cancers. Since then, multiple *Drosophila* models have been generated to further characterize the mutation and a possible mechanism of toxicity with relation to ALS.

A model of transgenic flies expressing ALS-mutant hFUS in various subpopulations of neurons showed age-dependent neurodegeneration, a characteristic of clinical and pathological features of FUS-ALS [53]. Moreover, flies generated through the UAS/gal4 system overexpressing mutant hFUS caused severe neurodegeneration in *Drosophila* eyes. A specific mutant, R521C, was associated with a decreased lifespan in flies [54]. Both of these studies have found that hFUS mutants express further degeneration than WT FUS [53,54].

Further studies examining the exact mechanism of disease have used *Drosophila* particularly in examining FUS’s relationship to the neuromuscular junction. In transgenic flies generated with the PhiC31 integration system that integrated hFUS or the *Drosophila* homolog in the *Drosophila* genome, it was found that FUS-related toxicity is dependent on the expression level of FUS in all examined tissues. It is proposed, due to morphological abnormalities found at the NMJ, that FUS causes toxicity by disrupting NMJs and possibly causing apoptosis in motor neurons [55].

This finding was furthered by a study done with flies overexpressing ALS-associated mutant FUS. These flies expressed decreased larval motility as well as changes at the NMJ. FUS expressing flies displayed an approximately 25% reduction in excitatory junction potential amplitude (EJP—a measure of the product of the quantal size and the quantal content) and was consistent with the findings in regards to VAMP mutations; it was found that the composition of postsynaptic glutamate receptors was altered with the overexpression of FUS in *Drosophila* motor neurons. More specifically, the reduction of quantal size associated with the reduction of EJP could be done by altering glutamate receptor clustering at the postsynaptic densities [56].

A more recent genetic screen compounded these results and identified a novel modifier of FUS. Using a transgenic fly line with FUS R521H mutations, it was found that knockdown of *muscleblind (Mbl)* in *Drosophila* was found to suppress wild-type and FUS mutant toxicity. This was accompanied by a gain of function approach that showed that flies overexpressing *Mbl* showed an enhancement of FUS toxicity. Consistent with previous findings regarding changes at the NMJ, it was found that knockdown of *Mbl* reduced the number of satellite boutons present in WT and mutant FUS [57].

Mislocalization of FUS has also been noted in mutants. WT hFUS distribution tends to be nuclear, while in ALS mutants, hFUS mislocalizes to the cytoplasm [53]. A genetic screen for modifiers associated with FUS-induced toxicity found that a nuclear pore protein, nucleoporin (Nup) 154, and the endogenous nuclear transport protein, Exportin1 (XPO1) suppressed FUS-induced neurotoxicity in *Drosophila* [27]. This could point to the integral role of nucleocytoplasmic transport proteins in FUS-toxicity.

A third, primary mechanism of toxicity with regards to FUS was developed using a *Drosophila* model. As mitochondrial dysfunction has been found in other genes connected to ALS, research in this area with regard to FUS has commenced, with interesting results so far. Flies expressing WT or P525L-mutant FUS in motor neurons were found to have significantly smaller axonal mitochondria in motor neurons than control flies, with mutants exhibiting more severe defects than WT FUS [28]. Compounded with cell line research showing that FUS is associated with the mitochondria, this suggests a possible model of toxicity through mitochondrial dysfunction [28].. *Drosophila* is a prime system to characterize the FUS mutation with regard to ALS and investigate possible models of toxicity.

Further modifiers and possible pathways of toxicity involving FUS have been identified utilizing *Drosophila*, including DDX17, a protein in the DEAD-box RNA Helicase family that is involved in RNA processing that is shown to be recruited into cytoplasmic stress granules in mutant FUS pathological conditions, thus inhibiting normal function [58]. FUS and various Nup-family protein interactions have also been shown to affect the nucleocytoplasmic transport through FUS mislocalization in a *Drosophila*-based study [59]. FUS is also shown in *Drosophila* to modify Hippo and JNK signaling, triggering cell death and neurodegeneration [60]. The variety in possible mechanisms of toxicity involving FUS is illustrated by a study with *Drosophila* done by [61].which elucidated that protein arginine methyltransferases 1 and 8 (PRMT1 and PRMT8) are associated with FUS. In *Drosophila* models of FUS-related ALS, loss of PRMT1 and PRMT8 exacerbate the degenerative phenotype, which indicates a possible role of arginine methylation in FUS induced toxicity.

## 8. C9orf72 and ALS—A Recent and Rapid Story

In 2011, a mutation was identified in ALS patients in the gene *C9orf72* (Chromosome 9 open reading frame 72). The gene’s toxicity has been linked to a GGGGCC (G_4_C_2_) hexanucleotide repeat expansion within its first intron. This mutation has been linked to a wide array of ALS patients and is the most common genetic factor associated with sALS noted so far. Thus, examining the model of toxicity of this repeat expansion is essential in understanding the disease.

The first model of this mutation in *Drosophila* was developed in 2013. Transgenic flies were developed expressing EGFP (a reporter gene), 3 G_4_C_2_ ([G_4_C_2_]_3_) repeats with EGFP, or 30 G_4_C_2_ ([G_4_C_2_]_30_) repeats with EGFP. The expression of EGFP alone or [G_4_C_2_]_3_-EGFP had no deleterious consequences, while the transient transfection expression of [G_4_C_2_]_30_-EGFP caused lethality in early development. Moreover, when [G_4_C_2_]_30_-EGFP expression was directed to the retina using GMR-gal4, severely disrupted eye morphology with various degrees of cell death, loss of pigmentation, and ommatidial disruption were noted. When expression of [G_4_C_2_]_3_-EGFP and [G_4_C_2_]_30_-EGFP was directed to the motor neurons, it was found that at day 28 post-eclosion, the [G_4_C_2_]_30_-EGFP expressed a significant reduction in locomotion [62].

Recently, further characterization of the repeat expansion on neuron morphology has been done in *Drosophila*. [G_4_C_2_]_48_ repeats expressed in the branched class IV epidermal sensory dendritic arborization neurons showed significant branching defects when compared to the control. These defects were compounded by age, with early third instar larvae expressing nearly normal branching while late instar larvae expressed a 42% decrease of distal intersections and a 53% loss of higher order branches, suggesting that the repeat expansion causes changes in neuron morphology [63].

A second model was developed in 2013 to investigate the exact mechanism of toxicity of the repeat expansion. A primary question in this investigation was whether the toxicity caused by this mutation was a result of the repeat RNA produced by bidirectional translation of the hexanucleotide repeat expansion or the dipeptide repeat (DPR) proteins generated by repeat-associated translation of the repeat RNA. *Drosophila* with “RNA-only” repeats were generated by inserting stop codons in all sense and antisense frames. These were then compared to flies with pure protein repeats, and it was shown that flies with pure repeats expressed eye degeneration, whereas RNA-only flies did not express this degeneration [29]. This indicates that the toxicity is likely caused primarily by DPR proteins, which was then further investigated by constructing “protein-only” flies using alternative codons to those found in the G_4_C_2_ repeat. When comparing two arginine-containing DPR proteins to two neutral proteins, flies with the arginine-containing proteins expressed lethality and eye degeneration was not found in flies expressing the neutral proteins [29]. Thus, a model can be proposed where basic arginine-containing DPR proteins cause toxicity of the repeat expansion in *Drosophila.*

A further model relating DPRs to a possible mechanism of toxicity was elucidated after it was found that transcripts encoding heat shock proteins (HSPs) regulated by the HSF1 transcription factor were found in C9orf72-ALS patients. It was found that flies expressing 49 G_4_C_2_ repeats in neurons showcased significantly increased expression of *Drosophila* orthologs of conserved C9orf72-associated HSPs and protein chaperones. These flies also expressed an increase in HSF1 expression similar to that found in human ALS patient brains, further showcasing *Drosophila*’s helpfulness in modeling human disease [64]. These data suggested the idea that HSF1 might be a potential modifier of C9orf72 toxicity. A fly line with an additional allele of dHSF1 was used to upregulate dHSF1 expression, which in turn enhanced G4C2 induced toxicity in the external eye [64]. This compounded with data showing that poly-GR_36_ (a specific DPR) external eye toxicity is enhanced in dHSF1 upregulation, suggests the model that HSF1 activity may enhance DPR-related toxicity in *Drosophila* [64].

Another pathway of pathogenesis has been introduced using *Drosophila* by Freibaum and colleagues. Using a model of DPR proteins, transgenic flies were constructed. These flies possessed a certain number of G_4_C_2_ repeats that had an open-reading frame for GFP to detect repeat associated non-AUG (RAN) translation associated with the production of DPRs. These flies expressed the dosage-dependent, repeat length-dependent degeneration and RAN translation of DPR proteins as observed in human patients, making this model viable. Using this model, a genetic screen identified 18 genetic modifiers that encode for parts of the nuclear pore complex and proteins involved in the export of nuclear RNA and the import of nuclear proteins, establishing nuclear transport defects as a mechanism of toxicity [65]. This was compounded by a genetic screen in a *Drosophila* model expressing 30 G_4_C_2_ repeats in the fly eye that identified a strong protein suppressor in an allele of RanGAP. This dominant, gain-of-function allele called RanGAP^SD^(GOF) suppressed the ommatidial disorganization defects that “normal” mutant G_4_C_2_ repeat flies expressed. Further adding to this, RanGAP knockdown-mediated enhancement of G_4_C_2_ degeneration worsens with age [30]. RanGAP stimulates Ran GTPase to hydrolyze GTP to GDP, which is an integral part of nucleocytoplasmic transport. Expressing carboxy-terminal HA-tagged *Drosophila* RanGAP protein in cells showed that RanGAP interacted with G_4_C_2_-repeat RNA in *Drosophila* cells, which suggested that the repeat expansion causes toxicity by interacting with RanGAP which alters nuclear transport. This was confirmed by expressing GFP with a classical NLS (Nuclear Localization Sequence) and an NES (Nuclear Export Signal) in the *Drosophila* salivary gland. In cells expressing G_4_C_2_, the ratio of NLS-NES-GFP is severely reduced when compared to WT *Drosophila* which suggests that nuclear import is inhibited and/or that nuclear export is enhanced [30]. These data in *Drosophila* suggest that nucleocytoplasmic transport defects are a possible mechanism of toxicity for the repeat expansion in C9orf72.

*Drosophila* have also been utilized to examine potential modifiers of C9orf72 toxicity. One such study by Ortega, et al. (2020) utilized *Drosophila* which expressed G_4_C_2_ repeats in the eye via GMR-GAL4 drivers to showcase the effects of eRF1, a protein that regulates nonsense-mediated decay (NMD). The *Drosophila* model showcased that reduction of eRF1 expression impaired motor function compared to controls while overexpression of eRF1 significantly suppressed eye degeneration, indicating that eRF1 could be a modulator of toxicity. Furthermore, *Drosophila* models expressing various lengths of G_4_C_2_ repeats showcased the effects of RNA-binding protein MATR3 to highlight its effects on C9orf72 pathology. *Drosophila* eye degeneration levels highlighted that deletion of the RNA-binding domain of MATR3 reduced the ability of MATR3 to suppress G_4_C_2_ toxicity, which showcases the potential role of MATR3 in the pathobiology of C9orf72-mediated ALS. *Drosophila* utilization has allowed for key mediator discovery in C9orf72 studies.

## 9. Drosophila’s Potential Pitfalls

As articulated throughout the review, *Drosophila* provides many advantages to studying neurodegeneration due to its simplicity and ease of manipulation. However, this simplicity is one of the major downsides of its use. Various *Drosophila* anatomy, including brain anatomy, differ drastically from humans, potentially influencing disease pathogenesis. It is also quite difficult to measure cognitive decline and differences in complex behavior with *Drosophila* [66] making studying neurodegenerative diseases specifically challenging. It is also worth recognizing that *Drosophila*, as an invertebrate, lacks complex features that may play a role in vertebrate neurodegeneration, including a less complex adaptive immune system that has been shown to be linked to ALS previously [67,68]. All of these aspects must be considered when researchers work in *Drosophila*; however, these pitfalls do not negate the versatility of *Drosophila* as a model system for studying ALS.

## 10. Future Research

Currently, many proposed theories regarding mechanisms of toxicity and pathogenesis of ALS-related genes need to be confirmed through further experimentation. *Drosophila* can continue to serve as a useful model for this, especially due to its ability to closely resemble the primary phenotypes of the disease. It also serves as a good tool due to its ease of genetic manipulation, short generation time, ease of care, and the large toolkit of genetic manipulations that can be utilized. Alongside confirmation of proposed theories, research is also continuing to focus on screening for genetic modifiers of ALS phenotypes. Many screens have revealed possible modifiers of phenotypic expression and introduced possible mechanisms of disease. Further screens must be done to uncover novel modifiers and help to confirm mechanisms of pathogenesis. Though *Drosophila*’s simplicity is what lends itself to its ease of use, it is not necessarily one hundred percent representative of mammalian pathogenesis. Thus, the data collected in *Drosophila* must also be confirmed in other models to establish these mechanisms and translate these findings to humans and develop treatments. As ALS research continues to expand, *Drosophila* can continue to serve as a versatile genetic tool to study the disease.

## Figures and Tables

**Table 1:** Various neurodegenerative diseases with their physiological characteristics and the use of *Drosophila* in studying them.

Disease Name	Characteristics	Examples of Uses of *Drosophila*	References
Huntington’s Disease	Motor and cognitive dysfunction, psychiatric symptoms	Used as a model to express toxic repeat expansion of *Htt* (Huntingtin) gene	[7]
	Known to be caused by an autosomal dominant repeat expansion in *Htt* gene	Used to study pathogenic *Htt* aggregates as a mechanism of pathogenesis	[8]
Parkinson’s Disease	Motor dysfunction including slow movements and tremors, and possible mild cognitive impairment and sleep disorders	Used to identify many genetic modifiers that might be involved in pathogenesis	[9]
	A variety of genetic factors have been linked, but no definitive cause	Used to model various forms of the disease including LRKK2-PD (Leucine Rich Repeat Kinase 2 – associated with Parkinson’s) and α-Syn-associated PD	[10]
	Associated with lower dopamine and norepinephrine levels and Lewy bodies	Used to study potential mutagens, including herbicides, effects on Parkinson’s	[11]
Alzheimer’s Disease	Disturbances in memory and language, impairment of higher executive functions A variety of genetic factors have been linked, but no definitive cause	Used to assess modifiers of the disease	[12]
	Accumulation of amyloid-beta plaques and tau tangles are known etiological signs	Used in modeling tau-associated toxicity, amyloid- beta-associated toxicity, and y-secretase models of toxicity (all common mechanisms of Alzheimer’s pathogenesis)	[13,14]

**Table 2:** The types of ALS and brief characteristics.

Familial ALS	Sporadic ALS	Other ALS-Associated Known Genes	Reference
~ 5–10% of ALS cases	~ 90–95% of ALS cases	NEK1 (NIMA Related Kinase I) – up to 3%	[17,18,19,20]
►Immediate family member has the disease	►No known close familial history of ALS	UBQLN2 (Ubiquilin-2)	
►Common genetic links include mutations in: c9orf72- up to 35% of cases SOD1- up to 15% of cases FUS- up to 5% of cases TDP-43- up to 4% of cases	►Common genetic links include mutations in: c9orf72- up to 5% of cases SOD1- up to 2% of cases FUS- up to 0.5% of cases TDP-43- up to 1% of cases	KIF5A (Kinesin Family Member 5A)	

**Table 3:** Common genes linked to ALS pathogenesis.

Gene	Role	Potential Pathways of Pathogenesis	Reference
SOD1 (Superoxide Dismutase I)	Responsible for getting rid of free superoxide radicals in the body via holding a Cu/Zn site for disproportionation of superoxide to hydrogen peroxide and dioxygen	− Oxidative stress on mitochondria ER	[21,22]
Alsin (*ALS2)*	GTPase regulator	− Endosomal trafficking dysfunction	[23]
VAP-B (VAMP (vesicle-associated membrane protein) protein B)	Involved in the unfolded protein response, vesicle trafficking, mediation of ER to Golgi tethering	− Endosomal trafficking dysfunction via failure to tether ER to Golgi − Failure of autophagic lysosomal degradation − Mitochondrial dysfunction	[22,24]
TDP-43 (TAR DNA-binding protein 43)	DNA/RNA binding protein that has been linked to transcription repression, pre-mRNA splicing, and DNA repair of double- stranded breaks	− Autophagy dysfunction − Oxidative stress on mitochondria	[25,26]
FUS (Fused in sarcoma)	RNA binding protein linked to transcription activation and DNA repair	− Nucleocytoplasmic Transport Defects − Mitochondrial dysfunction	[27,28]
*C9orf72*	Gene encoding a hexanucleotide repeat expansion mutation	− Toxic DPRs and RNAs − Nucleocytoplasmic transport defects	[29,30]

## Data Availability

Not applicable.
